# Improving complex agronomic and domestication traits in the perennial grain crop intermediate wheatgrass with genetic mapping and genomic prediction

**DOI:** 10.1002/tpg2.20498

**Published:** 2024-08-28

**Authors:** Prabin Bajgain, Hannah Stoll, James A. Anderson

**Affiliations:** ^1^ Department of Agronomy and Plant Genetics University of Minnesota Saint Paul Minnesota USA

## Abstract

The perennial grass *Thinopyrum intermedium* (intermediate wheatgrass [IWG]) is being domesticated as a food crop. With a deep root system and high biomass, IWG can help reduce soil and water erosion and limit nutrient runoff. As a novel grain crop undergoing domestication, IWG lags in yield, seed size, and other agronomic traits compared to annual grains. Better characterization of trait variation and identification of genetic markers associated with loci controlling the traits could help in further improving this crop. The University of Minnesota's Cycle 5 IWG breeding population of 595 spaced plants was evaluated at two locations in 2021 and 2022 for agronomic traits plant height, grain yield, and spike weight, and domestication traits shatter resistance, free grain threshing, and seed size. Pairwise trait correlations were weak to moderate with the highest correlation observed between seed size and height (0.41). Broad‐sense trait heritabilities were high (0.68–0.77) except for spike weight (0.49) and yield (0.44). Association mapping using 24,284 genome‐wide single nucleotide polymorphism markers identified 30 main quantitative trait loci (QTLs) across all environments and 32 QTL‐by‐environment interactions (QTE) at each environment. The genomic prediction model significantly improved predictions when parents were used in the training set and significant QTLs and QTEs used as covariates. Seed size was the best predicted trait with model predictive ability (*r*) of 0.72; yield was predicted moderately well (*r* = 0.45). We expect this discovery of significant genomic loci and mostly high trait predictions from genomic prediction models to help improve future IWG breeding populations.

## Core Ideas

- An advanced intermediate wheatgrass breeding population was evaluated for agronomic and domestication traits.
- A genome‐wide association scan identified a total of 62 significant genomic loci associated with the traits.
- Using the best two quantitative trait loci as covariates in a genomic prediction model slightly improved trait predictions.
- Including parents of breeding population in the training set significantly improved predictions for some traits.

**Abbreviations**
BLUE: best linear unbiased estimate
GEBV: genome‐estimated breeding values
GS: genomic selection
GWAS: genome‐wide association scan
IWG: intermediate wheatgrass
LD: linkage disequilibrium
QTE: QTL‐by‐environment interaction
QTL: quantitative trait locus
SNP: single nucleotide polymorphism
UMN: University of Minnesota

## 1 INTRODUCTION

Humans have been practicing crop domestication for thousands of years. Lately, several perennial crops have been selected as candidates for neo‐domestication and are being improved to produce grain and other plant products for food and feed. Examples include intermediate wheatgrass (IWG) and perennial forms of wheat, rice, sorghum, and sunflower, to name a few (Crews & Cattani, 2018). One remarkable benefit of cultivating perennial crops is their ability to remain productive for multiple cropping seasons with reduced farm inputs while delivering substantial agricultural outputs and adding less stress to the environment (Christian et al., 1997; Hoffman et al., 1995; Rogers et al., 2014).

IWG (*Thinopyrum intermedium* (Host) Barkworth & D.R. Dewey subsp. *intermedium*, 2*n* = 6*x* = 42) is one such novel perennial grass species currently being domesticated as a new food crop (Bajgain et al., 2022; Wagoner, 1990). Introduced to North America from Eastern Europe in the 1930s as a forage crop (Tsvelev, 1984), IWG is being improved for agronomic and domestication traits at multiple breeding programs within and outside the United States (Bajgain et al., 2022). The IWG breeding program at the University of Minnesota (UMN) was initiated in 2011 and has since completed six breeding cycles with one commercial variety released (Bajgain et al., 2020). Breeding progress throughout these cycles has resulted in germplasm with improved agronomic traits such as yield, plant height, and disease resistance, and domestication traits such as shatter resistance, free grain threshing, and larger seed size. Despite the improvements made in trait enhancement, IWG is still in its early domestication stages and needs further progress for it to be successful as a grain crop. Further improvement of IWG germplasm could be accelerated by applying modern genetic and genomic tools, for example, DNA marker discovery and genetic characterization of traits in the breeding populations.

Genetic characterization of traits of interest is generally done by associating genomic loci with the traits, and in the case of quantitative and complex traits, these loci are called quantitative trait loci (QTLs). As trait performances are subject to the environment where a plant population is under evaluation, statistical analyses are also conducted to assess QTL‐by‐environment (QTE) interactions to account for the effects of the environment (Lowry et al., 2019). Genome‐wide association scan (GWAS), also known as association mapping, is one common method used in discovery of QTL where genome‐wide DNA markers associated with traits are uncovered through relationships among trait distribution, allele frequency, and linkage disequilibrium (LD) (Weir, 2008). Single nucleotide polymorphisms (SNPs) are the overwhelmingly preferred DNA marker system for GWAS lately. This is because SNPs are abundant in genomes, offer excellent discriminatory power compared to nearly all other marker types, are relatively easy to convert to wet‐lab assays, and are mostly free of ascertainment bias (Bajgain et al., 2016, 2020). In mapping studies, the significance of these associations could potentially confound with allelic frequency due to population substructure. It therefore is often necessary to account for population structures existent in the population to avoid false associations (Vilhjálmsson & Nordborg, 2013). One approach to correct for population structure is to use genetic relationship among the genotypes being studied as a cofactor in the mapping model (Yu et al., 2005).

Identification of markers in strong linkage with the trait locus, especially those that explain a larger proportion of observed phenotypic variation, can potentially be used in marker‐assisted selection of the ideal genotypes, and also in discovery of candidate genes (Korte & Farlow, 2013). An improved understanding of the genetics behind important traits in IWG will be important in fast‐tracking the improvement of IWG germplasm. One method to achieve this is by studying the genetic variations behind these important traits, uncovering genetic loci controlling the traits, and their use in a recurrent selection scheme to select superior genotypes. Besides marker‐assisted selection, genomic selection (GS) is another tool that is increasingly being used in plant breeding programs to improve traits and populations (Voss‐Fels et al., 2019). In IWG breeding programs, GS is routinely implemented to improve the breeding populations and has been shown to be successful in accelerating the domestication rate of this crop (Bajgain et al., 2022).

This study was therefore carried out with two primary objectives: (1) characterization of genetic control of plant height, spike weight, grain yield, shatter resistance, free grain threshing, and seed size in the Cycle 5 IWG breeding population at the UMN and (2) assessment of the level of genetic variability present in the Cycle 5 IWG breeding germplasm by evaluating population structure, LD, and trait attributes such as distribution, correlation, and heritabilities. The potential benefit of using significant markers detected by QTL mapping models as covariates (i.e., fixed effects) in genomic prediction models in order to improve trait predictions was also explored.

## 2 MATERIALS AND METHODS

### 2.1 Plant population

The plant population used in this study is the fifth recurrent selection cycle (UMN‐C5 hereafter) of the UMN's IWG breeding program. This population of 595 genets was generated from open‐pollinating 40 Cycle 4 (UMN‐C4) genotypes that were selected based on the best genome‐estimated breeding values (GEBVs) (Bajgain & Anderson, 2021). The GEBVs were estimated from genomic prediction models trained using field data collected on the UMN‐C4 population in 2019 and 2020. These 40 parents were selected primarily for larger seeds, lower seed shattering, and improved grain threshing (threshability hereafter); strong selection was also applied for higher grain yield and short plant height. Parental plants were vernalized by placing them in a 4°C chamber for 8 weeks during October–December 2019 and crossed by open‐pollinating in a greenhouse during January–March 2020. Each mother plant was harvested individually and seeds were germinated in June 2020. After enough vegetative growth of the seedlings, all UMN‐C5 plants along with the 40 parents were cloned into two replicates in August 2020 and each set of clones was transplanted at two field sites in September 2020: Lamberton, MN, and Saint Paul, MN. Plants were transplanted with an inter‐plant distance of 0.91 m and the field was surrounded on all sides by a border of IWG bulk seed. Fields at both locations were fertilized with 50 kg ha^−1^ of N (urea) in April 2021 and 2022. The herbicide Dual II Magnum (S‐Metolachlor 82.4%, Syngenta) was applied in April of both years at both locations at a rate of 1.2 L ha^−1^. Additional weed control during the summer season was done with mechanical cultivation and manual labor. After the grain was harvested in August, plants were mowed to a height of 15–20 cm.

**Core Ideas**

- An advanced intermediate wheatgrass breeding population was evaluated for agronomic and domestication traits.
- A genome‐wide association scan identified a total of 62 significant genomic loci associated with the traits.
- Using the best two quantitative trait loci as covariates in a genomic prediction model slightly improved trait predictions.
- Including parents of breeding population in the training set significantly improved predictions for some traits.

### 2.2 Genotyping

Genomic DNA of each plant was extracted from 10‐ to 15‐cm long leaf tissue using the BioSprint 96 DNA Plant Kit (QIAGEN) and normalized to 10 ng µL^−1^. Sequencing libraries were double‐digested with the enzymes *PstI* and *MspI* following Poland et al. (2012) and sequenced on Illumina's Novaseq 6000 at the UMN Genomics Center. Obtained reads were filtered for a minimum quality (*Q*) of 30, de‐multiplexed, and aligned to the IWG reference genome v3.1 (Thinopyrum Intermedium Genome Sequencing Consortium, n.d.) using Burrows Wheeler Aligner 0.7.5a (H. Li & Durbin, 2009). Variants, mainly SNPs, were called using Samtools 1.6 and Bcftools 1.6 (H. Li et al., 2009). The SNPs were filtered to remove those with minor allele frequency (MAF) <3% and >10% missing allelic information; this led to a set of 24,284 genome‐wide SNPs. This set of SNPs was imputed with the LD‐kNNi method (Money et al., 2015) using 30 nearest neighbors in Tassel 5.2.93 (Bradbury et al., 2007). Imputed genotype data are available as Table S1.

### 2.3 Population structure and LD

Any existent population structure was analyzed using principal component (PC) analysis in Tassel 5.2.93 and visualized in R. LD among the markers was also calculated in Tassel with a sliding window of 50 markers. Resulting LD (*r* ^2^) values were plotted against physical distance of the IWG v3.1 reference genome and the distribution was fitted with a locally weighted polynomial regression (LOWESS) curve to display the LD decay trend. Distance of LD decay was calculated using the method proposed by Hill and Weir (1988) and assessed at an arbitrary *r* ^2^ value of 0.2.

### 2.4 Trait assessment and data analysis

The UMN‐C5 IWG breeding population was evaluated for several agronomic and domestication traits at two Minnesota locations in 2021 and 2022: Lamberton and Saint Paul. In this study, we focus our analysis on six important traits: three agronomic traits—(1) plant height, (2) spike weight, (3) grain yield—and three domestication traits: (4) shatter resistance, (5) threshability, and (6) seed size measured in terms of 1000‐kernel weight (TKW). Trait assessment procedure for each trait is described below:

1. Plant height (cm): The length of the tallest inflorescence (spike) from the ground; measured approximately 7 days before the plants were harvested.
2. Spike weight (g): Dry weight of one mature spike, measured by weighing the spike after drying at 32°C for 72 h. Three spikes per plant were weighed and averaged.
3. Grain yield (g): Weight of all grain produced per plant, measured after threshing the dry spikes on a Wintersteiger LD 350 (Wintersteiger Inc).
4. Shatter resistance: Tendency of spikelets, florets, and rachis to break off from dry spikes; measured on a scale of 0–9 where 0 means no breakage and 9 means 90% or more breakage. This was measured by applying physical force manually on three spikes per plant and values were averaged.
5. Threshability: Percentage of de‐hulled or naked grain after mechanical threshing of the mature and dry spikes on a Wintersteiger LD 350 to obtain grain. Threshability was measured on a scale of 0–9 where 0 is completely hulled grain and 9 is 90% or more de‐hulled grain.
6. Seed size (TKW, g): Weight of 1000 de‐hulled seeds. For samples with completely hulled grain post‐threshing, a wheat head thresher (Precision Machine Co., Inc.) was used to thresh and de‐hull the seeds.

Grain yield measurement could have been affected to a degree in case of breakage of dry, mature spikes in the field. An account of such scenario was not recorded because of resource constraints. Phenotype data discussed in this study are available as Table S2.

Broad‐sense heritabilities (*H* ^2^) for the traits were calculated on a genotype‐mean basis using the following equation:

$$H^{2}={\sigma_{g}}^{2}/\left({\sigma_{g}}^{2}+{\sigma_{gl}}^{2}/l+{\sigma_{gy}}^{2}/y+{\sigma_{e}}^{2}/ly\right),$$

where *σ*_g_^2^ is the genetic variance, *σ*_gl_^2^ is the genotype × location variance, *σ*_gy_^2^ is the genotype × year variance, *σ*_e_^2^ is the residual variance, *l* is the number of locations, and *y* is the number of years.

Trait data were corrected for environmental variability (i.e., trial effect) and the best linear unbiased estimates (BLUEs) for each genotype were obtained by using a mixed model in the R package “lme4” (Bates et al., 2015). Specifically, each environment was considered a fixed effect in the model and the effect value was removed for the traits in each environment to obtain the BLUEs for each genotype. Significant differences in group means of data distributions were determined using Tukey's honestly significant difference test at *α* = 0.05 level using the R package “agricolae” (de Mendiburu, 2020).

### 2.5 Genome‐wide association analysis for QTL discovery

For all traits, a search for genome‐wide QTLs was done using the BLUEs. The GWAS was performed using the “FarmCPU” method within the R package “GAPIT” in R 4.3.2 (Lipka et al., 2012). To control potential population structure during the GWAS, up to 10 PCs were evaluated using the “Model.selection” option. Model selection output suggested that no PCs should be used in the GWAS model (Table S3), therefore population structure was not controlled in the final GWAS. A Bonferroni correction method with a threshold at *α*  =  0.05 was used to declare significant QTL. This value equals to *α*/no. of markers  =  0.05/24,284  =  *p* value of 2.06 ×10^−6^ or logarithm of odds equivalent of 5.69. The amount of phenotypic variation (*R* ^2^) explained by the significant markers was estimated using the method of Sen and Churchill (2001) as implemented in the R package “qtl” (Broman et al., 2003).

In addition to identifying main QTL associated with the traits, a multi‐environment module was implemented to detect QTEs. For this purpose, we used the three‐variance multi‐locus random effect mixed linear model (IIIVmrMLM) (M. Li, Zhang, Xiang, et al., 2022; M. Li, Zhang, Zhang, et al., 2022). The multi‐environment analysis method (“Multi_env”), a multi‐locus GWAS model that estimates environmental interaction effects, was used with the parameters “SearchRadius = 20 and svpal = 0.001”. The *p* value threshold to declare significant QTEs was the Bonferroni threshold of 2.06 ×10^−6^.

### 2.6 Genomic prediction of agronomic and domestication traits

A genome‐wide trait prediction of traits (i.e., BLUEs) evaluated in this study was done using the R package “rrBLUP” (Endelman, 2011). Specifically, markers significantly associated with the six traits were used as covariates (fixed effects) in the prediction model. For each trait, seven scenarios with the following covariates were tested: (1) no SNP markers used as covariates, (2) the best two QTLs, that is, QTL that explained the largest and second‐largest observed phenotypic variation (*R* ^2^), (3) the best two QTEs, (4) the best two QTLs and QTEs, (5) all QTLs, (6) all QTEs, and (7) all QTLs and QTEs. In all scenarios, a four‐fold cross‐validation was implemented where 75% of UMN‐C5 was used as training set and the remaining 25% used as the validation set. The model was run for 100 iterations with four‐fold cross‐validation sets randomized in each iteration. Model performance was assessed by correlating the model‐estimated trait values with trait values collected in the field, that is, predictive ability (*r*).

## 3 RESULTS

### 3.1 Population genotyping and properties

A search for genome‐wide SNP markers by aligning the sequenced reads to the IWG reference genome v3.1 led to discovery of 11,462,673 SNPs in the UMN‐C5 population. After filtering the SNP markers to retain those with a minimum read depth of 5 or more, MAF of 3% or higher, and missing allele proportion of 10% or less, 24,284 markers remained. Chromosome S05 had the most number of markers (1644) and chromosome V04 had the least (640) with a genome‐wide mean of 1156. Imputation of missing alleles in Tassel using the LD‐kNNi method lowered the mean missing allele proportion from 2.9% to less than 0.1%. The post‐imputation mean population heterozygosity was 36% and 31% pre‐imputation.

**FIGURE 1:**
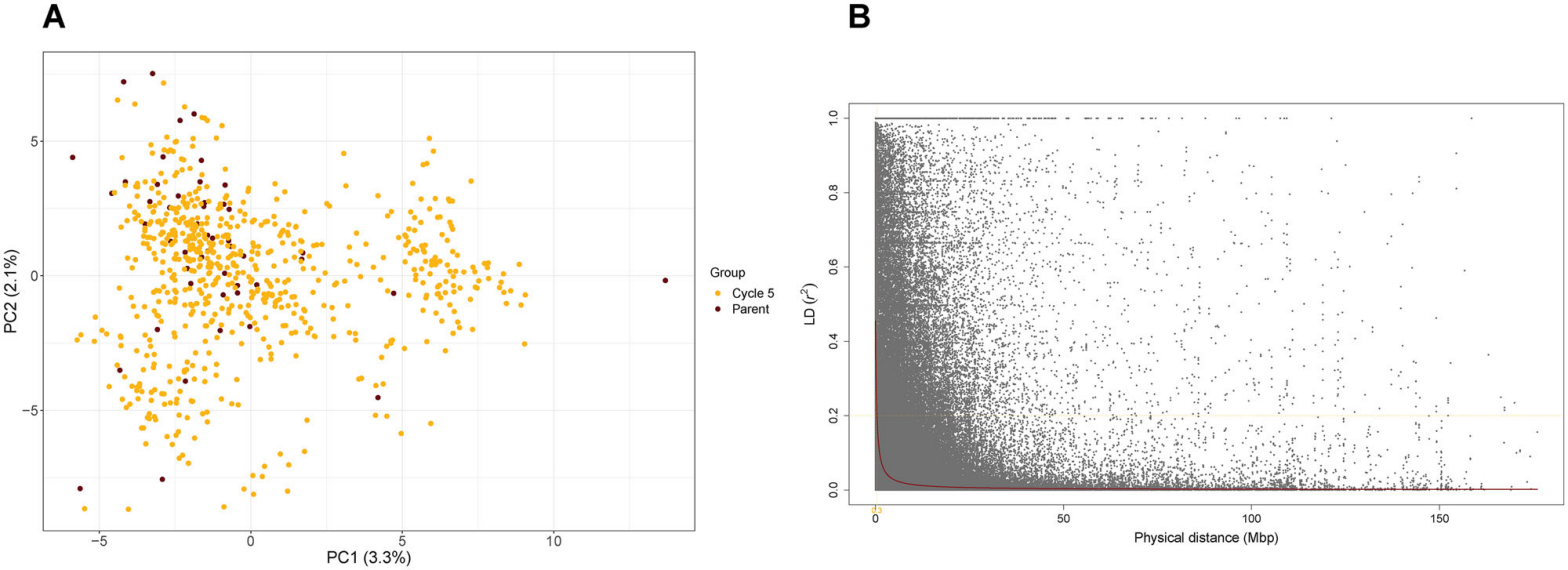
Visualization of population structure in the University of Minnesota (UMN)‐C5 intermediate wheatgrass breeding population by plotting principal component 1 (PC1) against principal component 2 (PC2) (Panel A). Panel B displays a genome‐wide linkage disequilibrium (*r* ^2^) values plotted against the physical distance in mega base pairs (Mbp) in the UMN‐C5 intermediate wheatgrass breeding population. A locally weighted scatterplot smoothing (LOWESS) curve in maroon color shows the decline of linkage disequilibrium (LD).

Population structure of UMN‐C5 was weak as the amount of genetic variation explained by the first two PC was 5.6% (Figure 1A); the first 10 PC axes explained approximately 17% of the total genetic variation. LD (*r* ^2^) varied largely among the 21 chromosomes from a minimum of 0.07 (Chromosome J04) to 0.10 mega base pairs (Mbp) (Chromosomes J07, S05, and V01), with a genome‐wide mean value of 0.09. The physical distance where LD reduced to half of its value at arbitrary yet conventionally reported *r* ^2^ = 0.20 was observed to be 0.30 Mbp in this population (Figure 1B).

### 3.2 Trait distribution, correlations, and heritability

**FIGURE 2:**
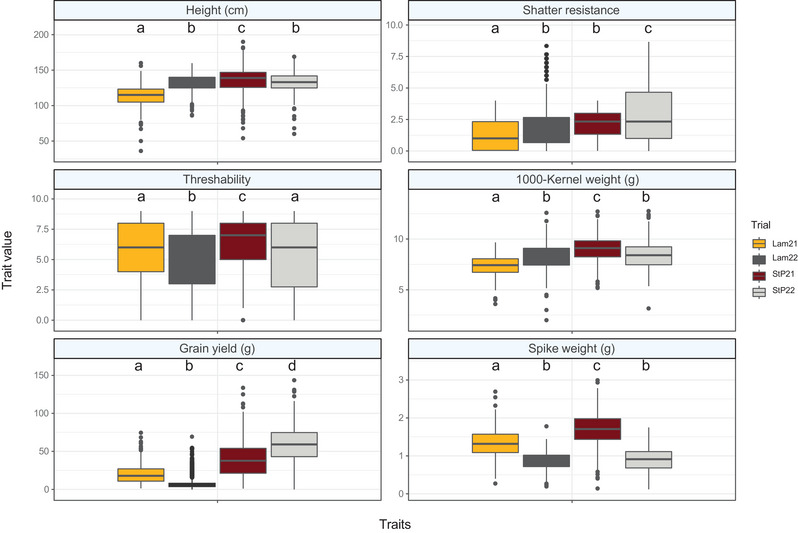
Distribution of trait data collected on the University of Minnesota (UMN)‐C5 intermediate wheatgrass breeding population. Data were collected at two Minnesota locations, Lamberton (Lam) and Saint Paul (StP), in 2021 and 2022. Common letters above the boxplots indicate that trait means are not significantly different by the Tukey's HSD (honestly significant difference) test at the significance level of *α* = 0.05.

First, an assessment of relationship between traits and the locations as well as the years where the populations were evaluated was done. Specifically, the significance of location and year effects on each trait was evaluated using an analysis of variance test. Relationships of all traits were significant with both location and year except for spike weight (location not significant) and TKW (year not significant; Table S4). Visualization of trait distribution revealed a wide range of quantitative trait variation in the UMN‐C5 IWG population for six agronomic and domestication traits: plant height, shatter resistance, threshability, seed mass (measured as TKW), grain yield, and spike weight (Figure 2). Across all four environments (i.e., locations × years combinations), a mean plant height of 129 cm was observed with the shortest plant measuring 36 cm (Lamberton 2021 environment) and the tallest plant measuring 190 cm (Saint Paul 2021 environment). Significant differences (at *α* = 0.05) in mean height were observed between all locations and years except for Lamberton 2022 and Saint Paul 2022 environments. The mean shatter resistance score was 2 (range of 0–9), and the second year plants were 31% more prone to shatter (lower shatter resistance) compared to the plants in the first year. The average threshability score was 6, and significant differences were observed between locations and years with the exception being Lamberton 2021 and Saint Paul 2022 that had similar population means. The smallest seed mass (TKW) was 2 g (Lamberton 2022) and the largest was 13 g (Saint Paul 2022) with an average of 8.3 g; the environment with the overall largest seeds was Saint Paul 2021 (average of 9 g). Grain yield in each environment was significantly different than the others. The largest grain yield per plant was observed in Saint Paul 2022 with a population mean of 59 g and the smallest yield was observed in Lamberton 2022 with a population mean of 10 g. Spike weights were also significantly different in all environments except for the second year trials in Lamberton and Saint Paul. The mean weight of a spike across all environments was 1.2 g and ranged from 0.3 to 3 g.

Pairwise correlation coefficients (*r*) for the six traits varied by trait pairs as they ranged from negative to positive values (Table 1). TKW (i.e., seed mass) and plant height had the strongest positive correlation at *r* = 0.41 followed by grain yield and plant height (*r* = 0.35), spike weight and shatter resistance, and grain yield and shatter resistance (*r* = 0.33 for both pairs). The strongest negative correlation was observed between shatter resistance and threshability (*r* = −0.15), followed by spike weight and threshability (*r* = −0.05). There was a moderate yet significant correlation between spike weight and TKW (*r* = 0.24). Weak and insignificant correlations were observed between threshability and TKW (*r* = 0.02).

**TABLE 1:** Pearson correlation coefficients (*r*) among the six agronomic and domestication traits across all four field trials in the University of Minnesota (UMN)‐C5 intermediate wheatgrass population.

Trait	Height (cm)	Shatter resistance	Threshability	TKW (g)	Grain yield (g)
Shatter resistance	0.08				
Threshability	0.09	−0.15			
TKW (g)	0.41^*^	0.20^*^	0.02		
Grain yield (g)	0.35^*^	0.33^*^	0.17^*^	0.14	
Spike weight (g)	0.19^*^	0.33^*^	−0.05	0.24^*^	0.32^*^

*Note*: Broad sense trait heritabilities (*H* ^2^) for all traits are presented in Table 2. The largest *H* ^2^ was observed for plant height (0.77), followed by threshability (0.76); the lowest observed *H* ^2^ was for grain yield (0.44).

Abbreviation: TKW, 1000‐kernel weight.

* Significance at *p* < 0.05.

## 4 GWAS RESULTS

A genome‐wide scan for associations between SNP markers and the six agronomic and domestication traits led to discovery of 30 significant QTLs in 15 chromosomes (Table 3). The number of QTLs ranged from one to five per chromosome. The greatest number of QTLs were discovered for spike weight (7), whereas height and yield had the least (four each). The QTL with the largest proportion of phenotypic variance (*R* ^2^) explained was observed for threshability (*R* ^2^  =  8.6% for marker *ChrJ04_334956931*). The average *R* ^2^ among all QTLs was 4.9% (median =  4.7%). For all six traits, QTL contributed both positive and negative allelic effects toward the traits. Both the largest and smallest allelic effects were observed for grain yield: 7.5 (23% of the mean yield across all locations and years), marker *ChrJ01_239578152* and −9.3 (28% of mean), marker *ChrJ02_627367414*. The average MAF of all significant SNP markers was 0.21. No significant SNP markers were common among the traits.

In addition to identifying main QTL associated with the traits, a three‐variance‐component multi‐locus random‐SNP‐effect mixed linear model, IIIVmrMLM, was implemented to detect QTEs. The model identified 32 QTEs distributed among 16 chromosomes (Table 4). The J genome had the highest number of QTEs (14) followed by V and S genomes (nine each). Spike weight had the highest number of QTEs (nine) and TKW had the lowest (four); no QTEs were observed for plant height. Overall, the QTEs explained a small proportion of observed phenotypic variance (*R* ^2^) as the mean *R* ^2^ for all QTEs was 1.8%; grain yield had the largest *R* ^2^ (4.4%) and threshability had the smallest (0.8%). There was no consistent pattern in allelic effects of these QTEs among locations and years. Yet, strong negative correlations were observed among a few location pairs (Table S5). The QTE loci for spike weight and grain yield—*ChrJ06_499783455* and *ChrJ06_500497650* respectively—were located within a distance of 0.71 Mbp, 0.11 Mbp larger than the genome‐wide distance where LD decayed to half its value at *r* ^2^ = 0.20.

## 5 GENOMIC PREDICTION

Results from genomic prediction of the six agronomic and domestication traits using a four‐fold cross‐validation model are displayed in Table 5. Most notably, including parents in the training dataset and using significant QTL and QTE as model covariates improved trait predictions by 1–11 percentage points. The largest improvement, of 11 percentage points, was observed for threshability when parents were part of the training set along with two best QTEs used as model covariates. Grain yield predictions improved by approximately 8 percentage points from 0.44 to 0.52. Improvements of 2–4 percentage points were still observed for all traits except plant height when parents were excluded from the training set but the two best QTLs and the two best QTEs were used as covariates in the prediction model. Using either two best QTLs or two best QTEs mostly improved predictions compared to a scenario where no QTL or QTE was used as model covariates, yet these varied by trait and were lower than the “Top Two QTL + QTE” scenario. Predictions when using all QTLs or all QTE or all QTLs and QTE were near‐identical with insignificant differences. Overall, TKW was the best predicted trait with a mean predictive ability (*r*) of 0.72 across all scenarios followed by shatter resistance (*r* = 0.69); grain yield (*r* = 0.45) was the most poorly predicted trait.

## 6 DISCUSSION

In this study, we evaluated the UMN's Cycle 5 IWG breeding population in 2021 and 2022 at two locations in the state of Minnesota: Lamberton and Saint Paul. Broad‐sense heritability estimates (*H* ^2^) for the traits across locations and years were moderate to high (Table 2). The lowest *H* ^2^ estimate was observed for grain yield (0.44) and largest for plant height (0.77) and threshability (0.76). The *H* ^2^ values observed in this study are typical of IWG populations as reported by previous studies and in different breeding programs (Bajgain et al., 2022). An exception is plant height, for which a higher degree of variability is observed in Minnesota as the state normally experiences cooler climatic conditions paired with an abundance in precipitation. Plant height *H* ^2^ estimates in Kansas and Utah, where other IWG breeding programs are located, are lower (ranging 0.38–0.47) compared to the value observed in our population (DeHaan et al., 2018; Larson et al., 2019). Yet, we acknowledge that these values have been obtained by evaluating genetically distinct populations at different locations and years, and thus should be interpreted with caution.

**TABLE 2:** Variance components and broad sense trait heritability estimates for the six agronomic and domestication traits plant height, shatter resistance, threshability, seed mass (measured as 1000‐kernel weight [TKW]), grain yield, and spike weight in the University of Minnesota (UMN)‐C5 intermediate wheatgrass population.

Trait	*σ*_g_^2^	*σ*_gl_^2^	*σ*_gy_^2^	*σ*_e_^2^	*H* ^2^
Height (cm)	121.91	24.32	15.22	66.43	0.77
Shatter resistance	1.04	0.15	0.06	1.54	0.68
Threshability	3.45	0.28	0.84	2.17	0.76
TKW (g)	0.60	0.04	0.11	0.77	0.69
Grain yield (g)	80.91	47.31	47.53	215.00	0.44
Spike weight (g)	0.94	0.06	0.41	2.90	0.49

We also observed moderate correlations among a few trait pairs, most notably the yield component traits having positive and significant correlations with plant height (Table 1). In previous studies, a similar relationship has been observed (Mortenson et al., 2019; Stoll et al., 2024; Zhang et al., 2016). Surprisingly, plants that performed better in yield component traits also had a higher tendency to shatter. A more concerning relationship, however, was between seed mass and grain yield (*r* = 0.14), which we expected to be higher because larger grain size generally contributes to higher grain yields, as we have observed in our prior populations (Bajgain et al., 2019). These deviations from past IWG breeding populations could be an anomalous relationship specific to UMN‐C5. These observations could also be attributed to environmental variables such as temperature and precipitation during the seasons when UMN‐C5 was grown and evaluated. The 2021 and 2022 summers had less rainfall at both locations: average of 2.0 and 2.7 mm during May–August in Lamberton and Saint Paul, respectively, compared to 3.2 and 3.7 mm at same locations between 2000 and 2020 (Table S6). Likewise, both locations were hotter than normal in 2021 and 2022 summers compared to the average values in the past 20 years. While these observations are informative, additional data are needed to make a definitive inference between changing environmental parameters and their effect on performance of IWG populations.

Prior to conducting the GWAS, a scan for population structure was done as the presence of population structures may lead to discovery of false marker‐trait associations (Lander & Schork, 1994; Yu et al., 2005). Results from PC analysis showed weak evidence for the presence of substructures in the UMN‐C5 population as the first two PC values explained <4% of the total genetic variation and <14% from the first 10 (Figure 1A). This low level of differentiation is not surprising as most likely only 20 parents were used to initiate the IWG breeding programs at UMN and elsewhere (Crain et al., 2024). The decay of LD (*r* ^2^) in UMN‐C5 was rapid, with *r* ^2^ decreasing to half of its value within a physical distance of 0.3 Mbp. This value is similar to what we have observed in our previous populations, with *r* ^2^ = 0.7 Mbp and 0.23 Mbp in Cycles 3 and 4, respectively (Bajgain et al., 2019; Bajgain & Anderson, 2021). Such short distances are not uncommon in outcrossing plants because of a high effective recombination rate (Wright et al., 2008). The pattern of LD decaying within a short genomic distance could also contribute toward more precision in QTL mapping as long as marker density is sufficient (Gaut & Long, 2003). A higher accuracy in mapping causative loci associated with traits of interest can assist in identifying tightly linked markers for marker‐assisted selection as well as in discovery of candidate genes.

A genome‐wide scan for six domestication and agronomic traits in the UMN‐C5 IWG breeding population led to discovery of 30 QTLs on 15 IWG chromosomes and 32 QTEs on 16 chromosomes associated with these traits (Tables 3 and 4). Within our study, no common SNP marker was shared among the main QTL and the QTEs. One possible reason could be because the genotype‐by‐environment interaction effects observed at each environment (location × year combination) were considered by the QTE detection model (M. Li, Zhang, Xiang, et al., 2022), whereas these effects were likely “diluted” when adjusted means were obtained by estimating BLUEs across all environments for the purpose of main QTL detection. We also compared the QTL detected in this study with previously published GWAS results in IWG. The number of previously published GWAS studies that have also used the v3.1 genome is limited, which constricts the breadth of our direct‐QTL comparisons. Of the five QTLs associated with shatter resistance in our study, *SS01_299017435* and *SS02_441397845* are located approximately 4.4 Mbp and 5 base pairs from the QTL *SS01_294528783* and *SS02_441397840*, respectively, reported by Crain et al. (2022), and thus could represent the same QTL. Other remaining QTLs reported in our study were not observed in previously published studies that used the v3.1 reference genome. With more studies in the future adopting the v3.1 reference genome, we expect more similar QTL regions among mapping studies.

**TABLE 3:** Significant quantitative trait loci (QTLs) associated with six agronomic and domestication traits in Cycle 5 intermediate wheatgrass (IWG) breeding population at the University of Minnesota: plant height, shatter resistance, threshability, seed mass (measured as 1000‐kernel weight [TKW]), grain yield, and spike weight.

Trait	SNP	Chr	Position (Mbp)	Alleles	MAF	*p* value	*R* ^2^ (%)	Allelic effect
Height	ChrJ02_410619409	J02	410.62	C/T	0.26	1.58E‐06	4.00	−2.81
Height	ChrS01_264749867	S01	264.75	G/T	0.25	6.12E‐07	4.27	3.15
Height	ChrS07_156573786	S07	156.57	G/A	0.08	6.40E‐07	4.26	4.93
Height	ChrS07_299891732	S07	299.89	C/T	0.34	2.59E‐07	4.53	−3.73
Shatter resistance	ChrS02_441397845	S02	441.40	G/A	0.32	2.09E‐08	5.26	−0.53
Shatter resistance	ChrJ07_582505014	J07	582.51	G/A	0.15	3.81E‐07	4.41	−0.47
Shatter resistance	ChrJ07_72064617	J07	72.06	C/A	0.43	2.68E‐07	4.52	−0.32
Shatter resistance	ChrS01_299017435	S01	299.02	G/A	0.22	3.85E‐08	5.08	−0.44
Shatter resistance	ChrV05_61388337	V05	61.39	A/G	0.12	4.16E‐09	5.72	0.46
Threshability	ChrJ04_334956931	J04	334.96	C/T	0.17	1.95E‐13	8.55	−0.95
Threshability	ChrJ05_318270505	J05	318.27	A/G	0.42	1.82E‐06	3.96	0.41
Threshability	ChrS02_72964609	S02	72.96	C/T	0.30	1.94E‐06	3.94	0.53
Threshability	ChrS02_405259988	S02	405.26	A/C	0.08	2.61E‐07	4.52	−0.91
Threshability	ChrS05_238563452	S05	238.56	A/C	0.34	9.34E‐08	4.82	0.36
TKW	ChrS03_80217631	S03	80.22	G/A	0.11	8.49E‐08	4.85	−0.51
TKW	ChrJ05_72516929	J05	72.52	G/A	0.12	1.59E‐07	4.67	0.40
TKW	ChrV03_311500128	V03	311.50	G/C	0.48	2.67E‐07	4.52	−0.25
TKW	ChrV04_376511108	V04	376.51	C/T	0.20	2.70E‐08	5.18	0.33
TKW	ChrV04_435561967	V04	435.56	C/G	0.34	2.93E‐07	4.49	0.26
Grain yield	ChrJ01_239578152	J01	239.58	C/T	0.05	6.68E‐07	4.25	7.52
Grain yield	ChrJ02_110660452	J02	110.66	G/A	0.41	1.06E‐10	6.77	−4.38
Grain yield	ChrJ02_465325394	J02	465.33	T/C	0.19	5.20E‐11	6.98	6.24
Grain yield	ChrJ02_627367414	J02	627.37	C/T	0.04	1.22E‐07	4.74	−9.33
Spike weight	ChrJ04_77782136	J04	77.78	G/A	0.04	1.62E‐06	3.99	1.15
Spike weight	ChrS05_331938937	S05	331.94	G/T	0.15	1.02E‐07	4.80	0.61
Spike weight	ChrJ02_642532185	J02	642.53	T/A	0.17	1.66E‐07	4.65	−0.56
Spike weight	ChrJ03_115931592	J03	115.93	C/T	0.17	1.53E‐07	4.68	0.58
Spike weight	ChrJ03_438671347	J03	438.67	C/T	0.15	1.64E‐07	4.66	0.55
Spike weight	ChrS05_395731228	S05	395.73	C/T	0.13	1.50E‐06	4.01	−0.57
Spike weight	ChrV06_87362741	V06	87.36	T/C	0.15	1.50E‐07	4.68	−0.56

*Note*: “MAF” is minor allele frequency of the marker and “*R* ^2^” is the proportion of phenotypic variance explained by the significant locus. QTLs are based on the BLUEs across locations for each trait as described in Section 2.

Abbreviation: SNP, single nucleotide polymorphism.

**TABLE 4:** Significant quantitative trait loci by environment (QTEs) associated with six agronomic and domestication traits in Cycle 5 intermediate wheatgrass (IWG) breeding population at the University of Minnesota: plant height, shatter resistance, threshability, seed mass (measured as 1000‐kernel weight [TKW]), grain yield, and spike weight.

								Allelic effect			
Trait	SNP	Chr	Position (Mbp)	Alleles	MAF	*p* value	*R* ^2^ (%)	Lam21	Lam22	StP21	StP22
Shatter resistance	ChrJ07_435826089	J07	435.83	A/G	0.07	4.23E‐09	1.0	0.00	0.25	0.00	−0.26
Shatter resistance	ChrS03_139619154	S03	139.62	C/A	0.26	6.50E‐08	2.4	0.27	−0.38	0.35	−0.24
Shatter resistance	ChrS05_531244737	S05	531.24	G/A	0.03	2.10E‐08	1.7	−0.04	0.31	0.06	−0.33
Shatter resistance	ChrS06_31476060	S06	31.48	A/G	0.04	8.64E‐08	1.6	0.16	0.22	−0.01	−0.36
Shatter resistance	ChrV02_493140713	V02	493.14	G/A	0.03	9.77E‐09	2.2	−0.06	−0.39	0.15	0.30
Shatter resistance	ChrV05_476664993	V05	476.66	C/T	0.44	8.22E‐10	3.1	−0.03	0.06	−0.03	0.00
Shatter resistance	ChrV07_201647999	V07	201.65	G/A	0.03	2.65E‐12	2.6	0.03	−0.40	−0.04	0.40
Threshability	ChrJ03_263754397	J03	263.75	C/T	0.10	2.62E‐09	1.0	0.03	0.34	−0.38	0.01
Threshability	ChrS05_310644630	S05	310.64	A/G	0.06	1.47E‐07	0.8	−0.10	0.36	−0.26	0.00
Threshability	ChrV02_211110818	V02	211.11	G/T	0.49	6.21E‐08	1.0	0.10	−0.42	0.40	−0.08
Threshability	ChrV06_318159807	V06	318.16	C/G	0.39	1.25E‐10	0.9	−0.05	−0.43	0.44	0.04
Threshability	ChrV07_261144173	V07	261.14	G/A	0.20	9.17E‐12	1.4	−0.15	0.29	−0.40	0.27
TKW	ChrJ01_449098323	J01	449.10	T/C	0.04	2.77E‐11	1.6	−0.04	−0.17	−0.06	0.27
TKW	ChrJ07_56579153	J07	56.58	G/T	0.06	5.00E‐08	1.0	−0.17	0.19	−0.02	0.01
TKW	ChrS05_416805119	S05	416.81	C/T	0.17	1.85E‐07	1.0	0.01	−0.20	0.02	0.17
TKW	ChrS06_54076849	S06	54.08	A/G	0.44	4.98E‐10	3.1	−0.04	0.01	−0.05	0.08
Grain yield	ChrJ04_136273585	J04	136.27	T/G	0.18	1.98E‐10	4.4	1.40	7.58	−4.23	−4.75
Grain yield	ChrJ05_254763334	J05	254.76	T/C	0.08	2.87E‐08	1.1	2.60	−3.54	0.66	0.29
Grain yield	ChrJ06_48752512	J06	48.75	T/A	0.23	7.45E‐11	1.9	−3.18	4.77	−2.96	1.36
Grain yield	ChrJ06_500497650	J06	500.50	G/A	0.08	2.49E‐08	1.1	3.59	−2.36	−0.04	−1.19
Grain yield	ChrS06_245946942	S06	245.95	T/C	0.09	2.97E‐07	1.0	−3.04	2.76	−0.22	0.50
Grain yield	ChrV04_82595423	V04	82.60	C/G	0.45	2.62E‐11	2.0	1.81	−0.13	1.57	−3.24
Grain yield	ChrV05_408125962	V05	408.13	A/C	0.26	3.35E‐10	3.4	2.46	0.96	−3.09	−0.32
Spike weight	ChrJ01_503139933	J01	503.14	G/A	0.04	5.42E‐08	1.7	0.25	−0.55	0.29	0.01
Spike weight	ChrJ02_102735299	J02	102.74	G/C	0.40	3.92E‐09	2.5	−0.11	0.15	−0.15	0.12
Spike weight	ChrJ03_374621658	J03	374.62	G/A	0.47	2.39E‐10	1.5	−0.14	0.61	−0.17	−0.31
Spike weight	ChrJ05_12245490	J05	12.25	A/G	0.13	5.81E‐10	1.9	0.02	0.22	−0.08	−0.16
Spike weight	ChrJ06_185133996	J06	185.13	G/A	0.21	4.26E‐08	2.0	0.37	−0.08	0.29	−0.57
Spike weight	ChrJ06_499783455	J06	499.78	C/A	0.40	5.31E‐09	2.7	−0.13	0.41	−0.37	0.09
Spike weight	ChrS01_346770788	S01	346.77	A/G	0.04	1.96E‐08	1.5	−0.16	0.53	−0.28	−0.09
Spike weight	ChrS06_167549694	S06	167.55	G/T	0.22	5.32E‐12	2.4	0.11	0.20	0.02	−0.33
Spike weight	ChrV06_356242527	V06	356.24	G/T	0.03	6.59E‐14	1.3	−0.01	0.46	−0.07	−0.37

*Note*: “MAF” is minor allele frequency of the marker and “*R* ^2^” is the proportion of phenotypic variance explained by the significant locus.

Abbreviation: SNP, single nucleotide polymorphism.

The IWG genome assembly was recently upgraded to version 3.1 from 2.1, which has led to changes in chromosome names and lengths, and rearrangement of sequences. While these changes have made QTL comparisons with previous studies difficult, we have made an attempt to identify QTL discovered on same chromosomes. For threshability, we observed five QTLs in four chromosomes of which three QTLs reside in chromosomes J04 near 335 Mbp (Chr10 in v2.1), J05 near 318 Mbp (Chr14 in v2.1), and S05 near 239 Mbp (Chr13 in v2.1). Previous reports of QTL associated with increased threshability have been reported near 183 Mbp of Chr10, 252 Mbp of Chr13, and 136 Mbp and 275 Mbp of Chr14 (Altendorf et al., 2021; Larson et al., 2019). For shatter resistance, five QTLs were detected in four chromosomes of which one QTL resides in chromosome S02 near 441 Mbp (Chr04 in v2.1). In a nested association mapping panel initiated from UMN's Cycle 1 parents, a QTL providing resistance to rachis breakage, a type of shattering, was found near 220 Mbp of Chr04 (Altendorf et al., 2021). Five QTLs in four chromosomes were found to be significantly associated with seed mass in our study. Of these, four QTLs reside in chromosomes J05 near 73 Mbp (Chr14 in v2.1), S03 near 80 Mbp (Chr08 in v2.1), V03 near 312 Mbp (Chr09 in v2.1), and 377 Mbp and 436 Mbp of chromosome V04 (Chr12 in v2.1). Bajgain et al. (2019), in their association mapping study conducted in the Cycle 3 IWG breeding population, reported QTL contributing to larger seeds near 122 Mbp of Chr08, 62 Mbp of Chr09, 88 Mbp of Chr12, and 327 Mbp of Chr14. For grain yield, we uncovered four QTLs in two chromosomes that were significant: J01 (Chr01 in v2.1) and J02 (Chr06 in v2.1). Other QTLs contributing toward higher grain yield were discovered near 426 Mbp of Chr01, and 30 Mbp and 373 Mbp of Chr06 in an association mapping study carried out by Bajgain et al. (2019). Likewise, of the seven significant QTLs discovered in our study, one was near 643 Mbp region in chromosome J02 (Chr06 in v2.1). There has been one report of a QTL associated with the trait spike weight near 373 region in their study (Bajgain et al., 2019).

All QTLs discovered in our GWAS explained small proportions of observed phenotypic distribution (*R* ^2^) as the mean *R* ^2^ across all QTLs was 4.9% and no *R* ^2^ value exceeded 9%. With similar values observed in previous IWG breeding populations, this is suggestive that selection of genotypes using markers alone may therefore be challenging in IWG. Instead, GS could be an alternative to improve these important complex traits as this approach estimates marker effects toward a trait in a population, which can then be aggregated to predict the performance of a genotype (Meuwissen et al., 2001). In previous work carried out by our breeding program as well as other IWG breeding programs, genomic prediction models have been shown to be effective in trait prediction and improving genetic gain while reducing breeding cycle time (Bajgain et al., 2022). Additionally, GS is also able to evaluate the effect of genome‐wide markers on multiple traits. This approach has been proposed to improve multiple traits simultaneously (Jannink et al., 2010). In a perennial crop such as IWG, phenotyping a large population for multiple years and locations can strain the resources available to a breeding program. GS will therefore remain as one of the central and most important tools implemented in IWG breeding programs to increase the rate of domestication of this species.

GS has been helping IWG breeding programs improve populations and accelerate domestication rate, and adding significant loci from GWAS as model covariates helped to improve trait predictions by two to four percentage points (Table 5). These values are different from what we have observed in our past study, that is, 2–4 percentage points improvement in this study compared to an improvement of 2–14 percentage points (Bajgain et al., 2019). However, this observation does corroborate the results observed in our past study where using SNP markers significantly associated with yield component traits in prediction models improved trait prediction. We observed a larger improvement in trait prediction when parents of the Cycle 5 population were included in the training set. Inclusion of parental data to improve trait prediction has previously been reported in other small grain species (Adeyemo et al., 2023; Montesinos‐López et al., 2023). In future populations, we expect the combined application of significant loci associated with traits of interest as well as the parents of the breeding population in genomic prediction models to boost trait predictions. Based on trait heritability estimates that are moderately high and diverse IWG breeding populations with good trait segregation, a considerable amount of genetic variation still exists in IWG breeding populations. This level of population diversity and genetic variation will continue to allow IWG breeders to impose strong selection pressure in future populations and increase the rates of trait domestication and genetic gain.

**TABLE 5:** Mean predictive abilities for six agronomic and domestication traits in Cycle 5 intermediate wheatgrass (IWG) breeding population at the University of Minnesota: plant height, shatter resistance, threshability, seed mass (measured as 1000‐kernel weight [TKW]), grain yield, and spike weight.

			Two best			All		
Trait	Scenario	No covariates	QTE	QTL	QTE + QTL	QTE	QTL	QTE + QTE
Plant height	No parents	0.57	0.58	0.58	0.58	NA	0.57	0.56
	With parents	0.58	0.61	0.61	0.62	NA	0.58	0.57
Shatter resistance	No parents	0.68	0.67	0.70	0.72	0.66	0.67	0.66
	With parents	0.69	0.71	0.75	0.75	0.67	0.68	0.67
Spike weight	No parents	0.59	0.61	0.60	0.63	0.59	0.58	0.59
	With parents	0.61	0.66	0.63	0.67	0.60	0.59	0.60
Threshability	No parents	0.54	0.52	0.56	0.58	0.52	0.52	0.53
	With parents	0.55	0.64	0.60	0.64	0.54	0.53	0.54
TKW	No parents	0.71	0.68	0.73	0.74	0.70	0.71	0.70
	With parents	0.72	0.72	0.76	0.78	0.71	0.72	0.71
Grain yield	No parents	0.43	0.43	0.44	0.48	0.43	0.42	0.43
	With parents	0.44	0.47	0.49	0.52	0.44	0.43	0.44

*Note*: As no QTEs were observed for plant height, the “QTL + QTE” values for this trait are predictive abilities obtained with only QTL as covariates in the prediction model.

Abbreviations: NA, not available; QTE, QTL‐by‐environment interaction; QTL, quantitative trait locus.

## 7 CONCLUSION

IWG is a perennial grain crop with potential to provide substantial ecosystem services and is currently being domesticated for grain production. The domestication timeline of this crop spans only a few decades with intensive breeding efforts dating back to the early 2000s. As the application of modern genomic tools to improve breeding germplasm is also recent, important traits in this crop still need characterization and significant improvements. In this study, we identified 30 QTLs and 32 QTEs associated with six agronomic and domestication traits: plant height, grain yield, seed size, shatter resistance, spike weight, and threshability. Using these significant loci as covariates along with the inclusion of parental data in genomic prediction models substantially improved predictions of a few traits. Discovery of these loci is expected to expand our current understanding of trait performance in IWG breeding populations.

## AUTHOR CONTRIBUTIONS

**Prabin Bajgain**: Conceptualization; data curation; formal analysis; funding acquisition; investigation; methodology; project administration; resources; software; validation; visualization; writing—original draft; writing—review and editing. **Hannah Stoll**: Data curation; writing—original draft; writing—review and editing. **James A. Anderson**: Funding acquisition; project administration; resources; software; supervision; writing—original draft; writing—review and editing.

## CONFLICT OF INTEREST STATEMENT

The authors declare no conflicts of interest.

## Supporting information

Supplemental Table S1 contains imputed genotype data for Cycle 5 IWG breeding population at the University of Minnesota.

Supplemental Table S2 contains phenotype data for six agronomic and domestication traits in Cycle 5 IWG breeding population at the University of Minnesota: plant height, shatter resistance, threshability, seed mass (measured as thousand kernel weight, TKW), grain yield, and spike weight.

Supplemental Table S3 contains the BIC values from GAPIT model selection results for each trait.

Supplemental Table S4 contains variance estimates for location and year effects for each trait.

Supplemental Table S5 shows Pearson correlation coefficients (r) among the allelic effects of the significant QTL by environment associations (QTEs) for six agronomic and domestication traits across all four field trials in the UMN‐C5 intermediate wheatgrass population.

Supplemental Table S6 has weather data for Lamberton, MN and St. Paul, MN between January 2000 and December 2022.

## Data Availability

Genotype data of UMN‐C5 IWG breeding population can be accessed through NCBI's Bioproject PRJNA1096721. Imputed genotype data are available as Table S1. Phenotype data are available as Table S2.
