# Supplementary material for: Improving complex agronomic and domestication traits in the perennial grain crop intermediate wheatgrass with genetic mapping and genomic prediction
Source: Plant Genome. 2024 Aug 28;18(1):e20498. doi: 10.1002/tpg2.20498 (PMC11726416; doi:10.1002/tpg2.20498)
Supplement: Supplementary file 1 [file TPG2-18-e20498-s003.docx]

**Supplemental Table 1**: Pearson correlation coefficients (*r*) among the allelic effects of the significant QTL by environment associations (QTEs) for six agronomic and domestication traits across all four field trials in the UMN-C5 intermediate wheatgrass population.

|  | **Lam21** | **Lam22** | **StP21** |
| --- | --- | --- | --- |
| **Lam22** | -0.43 |  |  |
| **StP21** | 0.07 | -0.80 |  |
| **StP22** | -0.51 | -0.39 | 0.26 |
