# Supplementary material for: Improving complex agronomic and domestication traits in the perennial grain crop intermediate wheatgrass with genetic mapping and genomic prediction
Source: Plant Genome. 2024 Aug 28;18(1):e20498. doi: 10.1002/tpg2.20498 (PMC11726416; doi:10.1002/tpg2.20498)
Supplement: Supplementary file 5 — Supplemental Table S5 shows Pearson correlation coefficients (r) among the allelic effects of the significant QTL by environment associations (QTEs) for six agronomic and domestication traits across all four field trials in the UMN‐C5 intermediate wheatgrass population. [file TPG2-18-e20498-s006.docx]

**Supplemental Table S5**: Pearson correlation coefficients (*r*) among the allelic effects of the significant QTL by environment associations (QTEs) for six agronomic and domestication traits across all four field trials in the UMN-C5 intermediate wheatgrass population.

|  | **Lam21** | **Lam22** | **StP21** |
| --- | --- | --- | --- |
| **Lam22** | -0.43 |  |  |
| **StP21** | 0.07 | -0.80 |  |
| **StP22** | -0.51 | -0.39 | 0.26 |
